# Diclofenac Hypersensitivity: Antibody Responses to the Parent Drug and Relevant Metabolites

**DOI:** 10.1371/journal.pone.0013707

**Published:** 2010-10-28

**Authors:** Andrea Harrer, Roland Lang, Robert Grims, Michaela Braitsch, Thomas Hawranek, Werner Aberer, Lothar Vogel, Walther Schmid, Fatima Ferreira, Martin Himly

**Affiliations:** 1 Division of Allergy and Immunology, Department of Molecular Biology, University of Salzburg, Salzburg, Austria; 2 Department of Dermatology, Paracelsus Medical University of Salzburg, Salzburg, Austria; 3 Department of Dermatology, Medical University of Graz, Graz, Austria; 4 Institute of Organic Chemistry, University of Vienna, Vienna, Austria; 5 Division of Allergology, Paul-Ehrlich-Institut, Langen, Germany; New York University, United States of America

## Abstract

**Background:**

Hypersensitivity reactions against nonsteroidal antiinflammatory drugs (NSAIDs) like diclofenac (DF) can manifest as Type I-like allergic reactions including systemic anaphylaxis. However, except for isolated case studies experimental evidence for an IgE-mediated pathomechanism of DF hypersensitivity is lacking. In this study we aimed to investigate the possible involvement of drug- and/or metabolite-specific antibodies in selective DF hypersensitivity.

**Methodology/Principal Findings:**

DF, an organochemically synthesized linkage variant, and five major Phase I metabolites were covalently coupled to carrier proteins. Drug conjugates were analyzed for coupling degree and capacity to crosslink receptor-bound IgE antibodies from drug-sensitized mice. With these conjugates, the presence of hapten-specific IgE antibodies was investigated in patients' samples by ELISA, mediator release assay, and basophil activation test. Production of sulfidoleukotrienes by drug conjugates was determined in PBMCs from DF-hypersensitive patients. All conjugates were shown to carry more than two haptens per carrier molecule. Immunization of mice with drug conjugates induced drug-specific IgE antibodies capable of triggering mediator release. Therefore, the conjugates are suitable tools for detection of drug-specific antibodies and for determination of their anaphylactic activity. Fifty-nine patients were enrolled and categorized as hypersensitive either selectively to DF or to multiple NSAIDs. In none of the patients' samples evidence for drug/metabolite-specific IgE in serum or bound to allergic effector cells was found. In contrast, a small group of patients (8/59, 14%) displayed drug/metabolite-specific IgG.

**Conclusions/Significance:**

We found no evidence for an IgE-mediated effector mechanism based on haptenation of protein carriers in DF-hypersensitive patients. Furthermore, a potential involvement of the most relevant metabolites in DF hypersensitivity reactions could be excluded.

## Introduction

Diclofenac (DF) is one of the most popular drugs world-wide belonging to the family of nonsteroidal antiinflammatory drugs (NSAIDs). Adverse drug reactions (ADRs), which occur in about every fifth patient, are well-known side effects mainly caused by the NSAIDs' acidic properties [1]. More serious are drug hypersensitivity reactions (DHRs) to DF, which clinically manifest as Type-I-like allergic reactions with sudden and unpredictable occurrence and can progress into anaphylactic shock. Two pathomechanisms are currently thought to be involved in NSAIDs hypersensitivity reactions: (i) multiple NSAIDs hypersensitivity (also those involving DF) has been considered as pharmacokinetic disorders due to non-selective coinhibition of cyclooxygenase (COX)-1 leading to shifts in the prostaglandin and leukotriene levels [2], [3]; (ii) in contrast, for selective NSAID hypersensitivities including DF, an involvement of the immune system is still being postulated [4]. Accordingly, drug-specific IgE has been reported in patients with DHRs to ASA, ibuprofen [5], propyphenazone [6], and sporadically to DF [7], [8]. Nevertheless, skin tests in DF-hypersensitive patients have persistently given negative results. Therefore, it is conceivable that various bioactivation and haptenation processes may play a role in DF hypersensitivity. However, adequate reagents for skin testing or *in vitro* assays are not available despite the fact that several Phase I metabolites of DF have been identified in human plasma, bile, and urine [9]. In addition, DF Phase II metabolites have been described in hepatocyte cultures [10]. The ability of Phase I metabolites to form immunogenic protein conjugates has been shown in animal studies [11], [12] and in cases of DF-induced immune hemolytic anemia [13]. Although protein conjugates of Phase II metabolites have not been described, it is possible that reactive intermediates of DF might haptenize to proteins in an alternative orientation.

In this study we performed a detailed investigation of drug-specific antibody responses in patients suffering from selective DHRs to DF. The possible role of drug-metabolism and bioactivation was examined using five major Phase I metabolites and one linkage variant of DF as haptens. The presence of drug/metabolite-specific IgE antibodies was investigated in serum and on basophils isolated from patients.

## Materials and Methods

### Ethics statement

The study was approved by the Ethics Committee for Experiments Involving Humans and/or Animals at the University of Salzburg complying with the Helsinki Declaraction as revised in 1983 and all patients participating gave their written informed consent.

Mice were maintained in the animal facility at the University of Salzburg and all animal experiments were conducted according to National guidelines approved by the Austrian Ministry of Science (BMWF-66.012/0011-II/10b/2010). The protocol was approved by the Ethics Committee for Experiments Involving Humans and/or Animals at the University of Salzburg. Blood drawals were done from the tail vein making all efforts to minimize suffering.

### Study population and clinical characterization

From 2005-2008, 59 patients (36 female, 23 male; mean age 53.9) with a history of acute hypersensitivity to DF (anaphylaxis I-IV, classification according to Ring and Messmer [14]) referred to the allergy centers of the university hospitals of Salzburg and Graz were studied. Patients underwent detailed clinical history and subjects with aspirin-exacerbated respiratory disease or chronic urticaria as underlying diseases were excluded. Characterization included skin tests, determination of total serum IgE and allergen-specific IgE against common aeroallergens (SX1, Phadia, Uppsala, Sweden) and 48 patients also underwent oral provocation testing (OPT). OPT was performed by a medical doctor in a single blind way with increasing dosage up to a usual single dose and administration intervals of at least 1.5 hours. The total dose was up to a normal daily dose. Patients were under continuous observation with emergency medication at hand and were told to report any occurring symptoms immediately. IgE measurements were carried out by ImmunoCAP™ with <100 kU/L total IgE considered normal and >0.35 kU/ml specific IgE considered positive. Six individuals (4 females, 2 males; mean age 38.7) who tolerated DF upon repeated administration within 2 years before analysis were included as controls.

### Hapten-carrier conjugates

DF sodium salt (Sigma, St. Louis, MO) or five Phase I metabolites (3′OH-DF, 4′OH-DF, 5OH-DF, 3′OH4′metO-DF, and 4′5diOH-DF, Novartis-Pharma, Basel, CH) were covalently coupled to human serum albumin (HSA) or keyhole limpet hemocyanin (KLH) using N-(3-Dimethylaminopropyl)-N'-ethylcarbodiimide hydrochloride (EDC) in a molar ratio protein:hapten:EDC of 1∶100∶100 for HSA conjugates and in a weight ratio of 1∶1∶0.25 for KLH conjugates.

A position-5 derivative of DF (DF5der) with a six C-atoms spacer containing an aldehyde group was synthesized as described [15]–[17]. Sodium cyanoborohydride (NaBH_3_CN, Sigma) was used for coupling DF5der to HSA in a molar ratio protein:hapten:NaBH_3_CN of 1∶20∶100 and to KLH in a weight ratio of 1∶0.3∶0.1. HSA-mock conjugates were produced analogously without addition of hapten and used as negative controls.

The coupling degrees of DF/DF5der/DF-metabolites to HSA (DF/HSA) were determined by High-Performance Size-Exclusion Chromatography (HPSEC) online coupled with a triple detector array (TDA302, Viscotek, Houston, TX, USA) as described elsewhere [18] with 100 µg conjugate on column. Detector was calibrated with HSA and values for dn/dc of 0.185 ml/g, dA/dc of 0.518 ml/g, and molecular weight of 66472 were set [19]. For all haptens dn/dc of 0.285 ml/g and dA/dc of 44.602 ml/g were determined from the quotients of refractive index (RI) and UV_280_ areas by the amounts DF injected. The concentrations of HSA and DF of eluting fractions were determined by following equations: RI area  =  k_RI_[(d*n*/d*c*)_HSA_*c_HSA_+(d*n*/d*c*)_DF_*c_DF_]; UV_280_ area  =  k_UV_[(d*A*/d*c*)_HSA_*c_HSA_+(d*A*/d*c*)_DF_*c_DF_].

### DF/DF5der/DF-metabolite-specific antisera

Female BALB/c mice (Charles River Laboratories, Wilmington, MA, USA) at 6 to 12 weeks of age were used to obtain polyclonal antisera. Animals were injected subcutaneously (s.c.) with with 5 µg DF/DF5der/DF-metabolite-KLH adsorbed to Alugel-S (Serva, Heidelberg, Germany) given as two 50 µl s.c. injections administered bilaterally in the lumbar region and boosted on day 14, 21, and 42. Sera were collected one week after a final boost and stored at −20°C until analysis.

### Mediator release assays

Release assay with mouse sera (mRBL): Beta-hexosaminidase release assays were performed with DF/DF5der/DF metabolite-specific mouse antisera (final dilution 1∶10) using RBL-2H3 cells (ATCC: CRL-2256™) and the respective HSA conjugates (varying concentrations), according to a protocol previously described [20].

Release assay with human sera (huRBL): Patients' sera were tested with the RBL-703/21 cells (optimized subclone of RBL-30/25 [21]), expressing the alpha-chain of the human high-affinity IgE receptor (FcεRI). HuRBL was performed as the mRBL assay with the following modifications: cell attachment (10^5^ cells/well) and passive sensitization with human sera (1∶10) were combined in an overnight (ON) incubation step and stimulation buffer contained 50% D_2_O (Sigma) for enhancement of release. Crosslinking was induced by addition of 1 µg/ml HSA conjugate. In patients with severe (grade III and IV) and immediate (<30 min) reactions crosslinking was measured for 12.5, 2.5, 0.25 µg/ml, 25, 2.5, 0.25 ng/ml HSA conjugate.

Cut-off was set at the mean percent release of negative controls (mock-HSA) plus 3 times the standard deviation (3*SD). Interassay performance of RBL-703/21 cells was controlled with serum from a birch-allergic donor (1∶10) and recombinant Bet v 1a (4 ng/ml). Possible nonspecific effects were controlled by stimulating cells with mock-HSA in both assay formats.

### ELISA

Plates were coated with 2.5, 1.0, and 0.25 µg/well DF/DF5der/DF-metabolite-HSA conjugates or mock-HSA. After washing and blocking, 50 µl patient sera (1∶2 and 1∶10) diluted in 25 mM tris-buffered saline pH 7.4, 0.05% Tween 20, 0.5% BSA were added and incubated (ON, 4°C). Extensive washing was followed by incubation (2 h, RT) with 50 µl alkaline phosphatase (AP)-labeled anti-human IgE (1∶2000, BD Pharmingen, Schwechat, Austria), mouse anti-human IgG1 (hinge)-AP or anti-human IgG4-AP (each 1∶1000, Southern Biotech, AL, USA). For detection, 100 µl 10 mM 4-nitrophenylphosphate in 9.7% (v/v) diethanolamine, 1 mM MgCl_2_, pH 9.5 were added and absorbance (405/492 nm) was measured after 0.5, 1, 2, and 3 h. Results were analyzed from 3 h measurements and cut-off was determined as mean absorbances plus 3*SD of mock-HSA. Values above background were considered positive when absorbance index (AI; absorbance test/absorbance individual negative control) >2, and when reproducible in two independent assays.

### Basophil activation test (BAT)

Basophil activation was performed from EDTA-whole blood using Flow2CAST (Bühlmann Laboratories, Schönenbuch, CH) according to the manufacturer's protocol. Whole blood samples were stimulated (20 min, 37°C) with HSA conjugates or mock-HSA at a final concentration of 1 µg/ml. Processed samples were immediately analyzed by flow cytometry (FACSCanto™ II, BD Bioscience, San Jose, CA) using FACSDiva 5.02. Basophils were gated as SSC^low^/CCR3^high^ and 500–1000 events recorded. Activated basophils were identified as CD63^high^ and cut-off was set with the negative stimulation control. Samples were considered positive at activation percentage >5% CD63^high^ cells and stimulation index (SI; % activated basophils divided by % activated basophils of the negative stimulation control) >2.

Cytotoxicity of DF/DF5der/DF-metabolite conjugates was investigated by costimulating samples with 1 or 20 µg/ml HSA conjugates plus 10 µg/ml anti-human IgE (20 min, 37°C). Basophil reactivity of the costimulated samples was calculated as percentage of activated basophils upon stimulation with anti-human FcεRI antibody taken as 100% anti-IgE reactivity.

### Cellular antigen stimulation test (CAST)

Antigen-induced production of sulfidoleukotrienes (sLTs) was investigated using CAST-2000-ELISA (Bühlmann Laboratories) according to manufacturer's instructions. Cells were stimulated with 1 µg/ml (final concentration) HSA conjugate or mock HSA. Supernatants were collected after short centrifugation and stored at −20°C until use. Samples were analyzed in duplicates and results calculated with the 4-parameter-curve-fit option of Deltasoft IIIv2.248 (BioMetallics, Princetown, NJ, USA). Sulfidoleukotrienes were considered significantly increased when above 163.8 pg/ml, a cut-off that was calculated from the mean plus 3*SD of negative stimulation controls (patient #54 excluded because of high background).

## Results

### Defining phenotypes of DF-hypersensitive patients

In this study, 59 patients with acute hypersensitivity to DF were recruited (Table S1). In order to exactly define the patterns of reactivity of patients towards DF and other NSAIDs 41 patients underwent OPTs with various NSAIDs, but not with DF, seven patients were challenged with DF, and for eleven patients definition of clinical reactivity was based on history alone. Accordingly, patients were divided into two groups: individuals reporting an intolerance reaction exclusively to DF or tolerating other NSAIDs in OPT were considered monosensitive to DF (DF patients, n = 41); patients reacting with other NSAIDs in OPT or reporting an intolerance reaction to NSAIDs were classified as multiple NSAIDs-reactive (NSAIDs patients, n = 18). 48% (20/41) of DF patients showed anaphylactic reactions with severity grades III and IV. Onset of symptoms was immediate (≤1 h) in 22/41 cases, accelerated (≤12 h) in 12/41, and not specified in 5/41. In contrast, 83% (15/18) of NSAIDs patients displayed severity grade I or II reactions. Non-immediate onset of symptoms was no exclusion criterium for this study as delays have been observed in verified IgE-mediated reactions and suggested to result from several factors including drug metabolism [22].

Skin test results were positive for three patients, two reacting exclusively with DF (DF patient #59, NSAIDs patient #55) and one with various NSAIDs (ASA, ibuprofen, metamizole, oxicams, NSAIDs patient #31) but not with with DF. Parameters of atopy showed no differences between DF and NSAIDs patients with increased levels (>100 kU/l) of total IgE in 29% and 35% and with specific IgE against inhalant allergens (SX1) in 22% and 20% of the patients, respectively.

### Physicochemical and functional characterization of hapten-HSA conjugates

**Figure 1:**
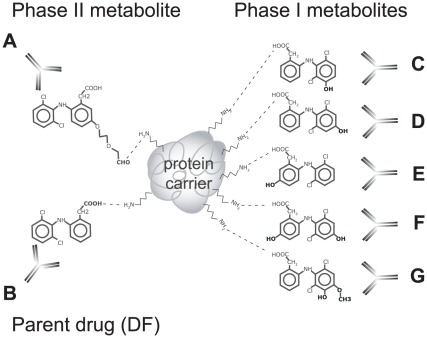
Coupling strategy. Bioactivation resulting in haptenation was mimicked by linkage of native DF (A) via position 5 or (B) via position 1 and linkage of five Phase I DF-metabolites (C) 3′OH-DF, (D) 4′OH-DF, (E) 5OH-DF, (F) 4′5diOH-DF, (G) 3′OH4′metO-DF via position 1 to HSA as protein carrier.

**Figure 2:**
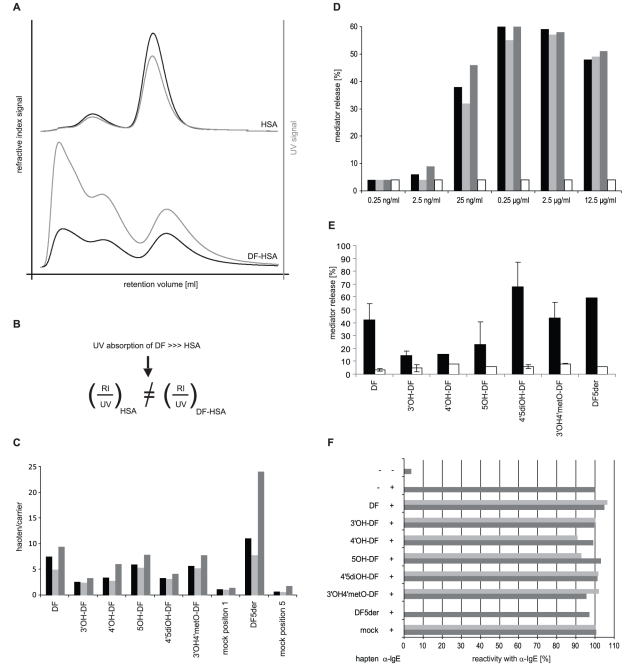
Characterization of hapten-HSA conjugates. (A) Coupling degree of eluting HPSEC fractions derived from hapten-HSA conjugates was determined from (B) the ratio of refractive index (black) to UV signal (gray) resulting in (C) several haptens per carrier molecules for all conjugates compared to respective mock controls; determined coupling degree for total, monomeric, and aggregated HPSEC fractions are illustrated in black, light, and dark gray, respectively. (D) Determination of optimal concentration of DF-HSA conjugate for FcεRI-crosslinking/mediator release; determined values for total, monomeric, and aggregated HPSEC fractions are illustrated in black, light, and dark gray, respectively, mock controls in white. (E) All hapten-HSA conjugates (dark gray) showed mediator release (n = 1–4) from rat basophil leukemia cells when incubated with sera from immunized mice; mock controls in light gray. (F) Investigation of cytotoxicity of hapten-HSA conjugates coincubated at 1 (dark gray) and 20 µg/ml (light gray) with anti-IgE by basophil activation test (100% without conjugate).

Human serum albumin was chosen for chemical conjugation of DF/DF5der/DF-metabolites (Figure 1) as it is the most abundant plasma protein and a physiological carrier of slightly acidic xenobiotics like DF [23]. Mock-HSA was used to control for a formation of neoepitopes, which might result from inter- and intramolecular crosslinkings. Antigenic bivalency as minimum theoretical requirement for a receptor cross-linking activity was given as coupling degrees were shown to exceed 2 DF/HSA in all conjugates as determined from the ratio of UV to RI signals from HPSEC (Figure 2A–C).

Allergenic functionality of the HSA conjugates was demonstrated by mRBL-assay using DF/DF5der/DF-metabolite-specific mouse antisera. HPSEC-derived fractions containing DF-HSA monomer (5.0 DF/HSA) or DF-HSA aggregate (9.5 DF/HSA) and unfractionated DF-HSA (7.6 DF/HSA) were analyzed in a dilution series from 0.25 ng/ml to 12.5 µg/ml. As shown in Figure 2D, FcεRI-crosslinking capacity of the DF-HSA conjugates was independent of aggregation state, a plateau was reached at 0.25 µg/ml, and the bell-shaped dose-response curve extended over >3 orders of magnitude. Further experiments were conducted with unfractionated conjugates only and 1 µg/ml was determined adequate (center of bell-shaped dose-response curve) to investigate IgE-mediated degranulation. All HSA conjugates had functional IgE receptor-crosslinking capacities at this concentration as shown in Figure 2E. Comparability of mediator release assays using RBL-2H3 and RBL-30/25 cells for determination of specific IgE in murine and human sera has been shown previously [24].

Potential cytotoxicity of DF/DF5der/DF-metabolite-HSA was controlled in BAT by whole blood basophils activated with 10 µg/ml anti-human IgE in presence of 1 or 20 µg/ml conjugate. Reactivity of basophils in costimulated samples was not affected compared to positive stimulation control (taken as 100% reactivity), showing that HSA conjugates were not cytotoxic (Figure 2F).

### Hapten-HSA conjugates as tools to detect drug-specific antibody responses

Four approaches were used to investigate antibody responses against DF and metabolites thereof. Antibodies in serum were determined by ELISA and mediator release assay. Fifty-nine patient sera and sera from six DF-tolerant controls were tested in ELISA for DF/DF5der/DF-metabolite-specific IgE, IgG_1_ and IgG_4_ antibodies. Only two sera showed IgE reactivities, one against DF-HSA and the other against DF5der-HSA. Surprisingly, one of these sera was from a DF-tolerant control. IgG-reactivities against DF-, 4′OH-DF-, 5OH-DF-, and 4′5diOH-DF-HSA could be observed in seven patients (five DF patients, two NSAIDs patients) and were mainly of the IgG_4_ subtype. Three of these patients with positive IgG results had a history of severe anaphylactic reactions. In one patient with grade IV reactions, IgG_4_ against DF-, 4′OH-DF-, and 4′5diOH-DF-HSA could be detected. No antibody reactivity could be detected for 3′OH-DF- and 3′OH4′metO-DF-HSA conjugates. Results of observed reactivities are summarized in Table 1.

**Table 1:** Summary of positive ELISA results of antibody reactivity against hapten-HSA conjugates.

ID	Reactive to	Grade	α-DF	α-DF5der	α-3′OH-DF	α-4′OH-DF	α-5OH-DF	α-4′5diOH-DF	α-3′OH4′metO-DF
#5	DF	IV	neg	neg	neg	neg	IgG1	neg	neg
#20	DF	III	neg	neg	neg	neg	IgG4	neg	neg
#25	DF	II	neg	IgE	neg	neg	neg	neg	neg
#42	DF	IV	IgG4	neg	neg	pos	neg	IgG4	neg
#46	DF	II	neg	neg	neg	IgG1, IgG4	neg	neg	neg
#50	DF	II	neg	neg	neg	IgG4	neg	neg	neg
#4	NSAID	I	neg	neg	neg	neg	neg	IgG4	neg
#23	NSAID	II	neg	neg	neg	IgG1	neg	neg	neg
CG02	control	0	IgE	neg	neg	neg	neg	neg	neg

ELISA results were considered positive exceeding the cut-off defined as the means of negative controls (mock-HSA) plus 3 standard deviations and an absorbance index >2. Only patients with any postive reactivity are listed.

Mediator release assay with RBL-703/21 cells was performed as this method may be more sensitive for the detection of low levels of drug-specific IgE antibodies. Sera from 39 patients (26 DF patients, 13 NSAIDs patients) including all three individuals with grade IV anaphylactic reactions were analyzed. Five sera had to be excluded and results of eight sera were ambiguous, as viabilities of huRBL cells were ≤20% and ≤45%, respectively. Upon stimulation with all hapten-HSA conjugates no β-hexosaminidase release above background (>9.5%) was observed with any of the tested patients′ sera (n = 26).

Reactivity of cell-bound antibodies was assayed by BAT and CAST in selected patients (i) with <2 years interval between hypersensitivity reactions and present study as sensitization to drugs was shown to be lost with time [25] and (ii) without immunomodulatory medication. Basophils from 19 patients (15 DF patients, four NSAIDs patients) and three DF-tolerant controls were analyzed by flow cytometry for surface-bound DF/DF-metabolite-specific IgE. Furthermore, basophils of all three selective DF-hypersensitive and provocation test-positive patients (#52, 53, and 58) were stimulated with the linkage variant, DF5der-HSA (Table S2). DF patient #20 was excluded from evaluation because basophils were not responsive to positive stimulation control, NSAIDs patient #54 because of high activation background (44.5%). DF/DF5der/DF-metabolite-HSA did not cause CD63 upregulation in basophils of the remaining 17 patients indicating lack of membrane-bound drug-specific IgE.

*De novo* production of sLTs was measured in supernatants of human peripheral blood mononuclear cells (PBMCs) from 19 patients (15 DF patients, four NSAIDs patients) and three DF-tolerant controls after stimulation with hapten-HSA conjugates (1 µg/ml). Only with PBMCs of DF patient #58 a slightly elevated sLT value of 194 pg/ml in response to 3′OH-DF-HSA was observed (Table S2).

## Discussion

Hypersensitivities caused by a single NSAID with concomitant tolerance to other NSAIDs clearly argue against a pharmacokinetic imbalance as the driving mechanism. Based on this assumption we sought to carefully define the phenotype of DF-hypersensitive patients in order to investigate the possible involvement of drug-specific immune responses. Due to the risk of anaphylaxis, in the present study we followed current recommendations [26] and did not perform double-blind placebo-controlled drug challenges. Exceptions involve a few patients who have been challenged with DF during their individual diagnosis procedure. In order to distinguish individuals selectively reactive to DF but tolerant to other NSAIDs (DF patients) from multiple NSAIDs-reactive subjects, patients were subdivided into two groups according to history and OPT with alternative NSAIDs. Interestingly, a higher percentage of DF patients displayed more severe (grade III/IV) reactions than the multiple NSAIDs-reactive group. Positive skin tests found in three patients from both groups confirmed the low predictive value of using unhaptenized drugs for diagnosis. In this respect, the need for *in vitro* diagnostics has been recently pointed out by the EAACI/ENDA task force for classification, diagnosis, and management of NSAIDs hypersensitivity [4].

As the observed specificity in DF patients is suspected to be immune-mediated, drug-specific antibody responses were investigated. Because haptenation and drug metabolism may play a role we used a panel of HSA conjugates of drug and metabolites. IgE in serum samples and bound to basophils was determined in DF and multiple NSAIDs patients, as well as in DF-tolerant controls. Specific serum IgE was not detected in hypersensitive patients, except for one DF patient showing marginal levels of DF5der-specific IgE in ELISA. Interestingly, IgG reactivity against DF, 4′OH-DF-, 5OH-DF-, and 4′5diOH-DF-HSA was observed in some individuals highlighting the immunogenic potential of the investigated molecules. This is in line with reports on formation of erythrocyte neoantigens and anti-4′OH-DF-specific antibodies in patients with DF-induced immunohemolytic anemia [12], [27] and reports on oxidized 5OH-DF as antigenic determinant for T cell proliferation in metabolite-immunized mice [11]. Thus, the importance of investigating bioactivation and metabolism in DF hypersensitivity is clearly demonstrated. Linkage of DF/DF-metabolites by their carboxyl groups (position 1) to HSA mimics a chemical modification of proteins via reactive acyl glucuronides, which is the most important mechanism of detoxification [10]. The glucuronidation process was shown to bioactivate drugs leading to covalent binding to proteins or formation of conjugates [28], [29]. Alternatively, linkage of DF via position 5 mimics potential drug-protein-conjugates resulting from reactive Phase II metabolic intermediates that escaped detoxification by glutathione [30], [31]. In this study, no evidence for DF/DF5der/DF-metabolite-specific serum IgE was found in ELISA or mediator release assay. However, these findings have not excluded all the possibilities of IgE-mediated reactions to DF yet. In fact, we observed that basophils from birch-allergic donors collected off-season were reactive in BAT, but the corresponding pollen-specific serum IgE was negative or borderline when determined by ELISA (data not shown). As basophils have been recently shown to be key players in the immune system [32], we investigated DF/DF5der/DF-metabolite-specific IgE bound on patients' basophils by BAT and sLT production. These methods analyze two consecutive cellular events in response to IgE-receptor crosslinking: upregulation of basophil activation markers due to degranulation followed by release of preformed mediators and production of sLTs as broncho- and vasoactive mediators of the allergic reaction. However, neither of these two methods provided convincing evidence for the existence of drug/metabolite-specific IgE antibodies.

This study represents the first comprehensive mechanistic investigation of a potential IgE-mediated mechanism in selective DF hypersensitivity. In addition to the drug itself the most relevant DF metabolites were examined. Two known DF metabolites were not included in this study. First, an alternative side product of the reactive DF acyl glucuronide, which was described for the NSAID tolmetin [33]. However, immunization of mice with tolmetin acyl glucuronide led to the production of antibodies cross-reactive to tolmetin [34], suggesting that this type of metabolites share relevant epitopes with the parental NSAIDs. Second, derivatives of reactive 2′,3′-oxide resulting in 2′-OH-monofenac linked to glutathione via position 3′, which were found in human liver microsomal preparations [35]. In principle, such metabolites might escape the glutathione detoxification process and form haptens, however, no protein conjugates have been described so far. It is noteworthy that mice immunized with DF or the five Phase I metabolites responded with IgE cross-reactive to all haptens included in this study (data not shown). Consequently, we are confident that the approach of the present study should allow detection of IgE specific for acyl glucuronides of DF as well as not yet identified structurally closely related DF metabolites.

In conclusion, our findings provide evidence that antibodies, in particular IgE, are not involved in DF hypersensitivity. It is possible that the clinical conditions of certain individuals at the time of medication might influence reactivity to the drug, adding another level of complexity to this pathology. This may include anaphylactic progression due to acute inflammation, potential subclinical diseases, interactions between medications, or even psychogenic effects. At present, a pathomechanism encompassing these effects remains speculative.

## Supporting Information

Table S1 Characteristics of the study group
(0.17 MB DOC)

Table S2 Combined results of BAT and CAST analyses
(0.13 MB DOC)
