# Supplementary material for: Diclofenac Hypersensitivity: Antibody Responses to the Parent Drug and Relevant Metabolites
Source: PLoS One. 2010 Oct 28;5(10):e13707. doi: 10.1371/journal.pone.0013707 (PMC2965666; doi:10.1371/journal.pone.0013707)
Supplement: Table S1 — Characteristics of the study group (0.17 MB DOC) [file pone.0013707.s001.doc]

**Table S1.** Characteristics of the study group

| ID | Sex | Age | Route | Sens | Grade | Clinical description | Onset | Inter | total IgE | SX1 | Skin | OPT | | History |
| --- | --- | --- | --- | --- | --- | --- | --- | --- | --- | --- | --- | --- | --- | --- |
|  |  |  |  |  |  |  |  |  | [kU/L] |  | SPT/other | reactive to | tolerant to | DHR |
| DF patients – single NSAID-reactive | | | | | | | | | | | | | | |
| *1* | *F* | *39* | *po* | *?* | *III* | *urt/hypo/coll* | *90 min* | *1 mo* | *65,1* | *nd* | *neg* |  | *ASA, AA, Ibu, Mef, Met, Ox* | *DF* |
| 2 | F | 45 | po | yes | II | dysp | 30 min | 1 wk | 52,5 | nd | neg |  | ASA, AA, Ind, Mef, Met, Ox | DF |
| *3* | *F* | *34* | *po* | *?* | *III* | *flush/naus/coll* | *10 min* | *>9 mo* | *32,2* | *nd* | *neg* |  | *AA, Ibu, Met, Ox* | *DF* |
| *5* | *M* | *66* | *iv* | *?* | *IV* | *urt/asth/shock* | *30 min* | *2 wk* | *8,12* | *nd* | *neg* | *nd* | *nd* | *DF* |
| *7* | *F* | *65* | *iv* | *?* | *III* | *prur/naus/coll* | *dur inf* | *3 wk* | *441* | *nd* | *neg* |  | *ASA, AA, Ibu, Mef, Met, Ox* | *DF* |
| *8* | *M* | *67* | *po* | *yes* | *III* | *urt/ang/coll* | *7 h* | *3 wk* | *>5000* | *nd* | *neg* | *nd* | *nd* | *DF* |
| 9 | F | 37 | po | ? | I | ang | --- | --- | 478 | nd | nd |  | ASA, AA, Ibu, Mef, Met, Ox | DF |
| 10 | F | 58 | po | yes | I | ang | 2 h | 5 wk | 53,8 | nd | nd |  | ASA, Ibu, Mef, Met, Ox | DF |
| *11* | *M* | *59* | *iv* | *yes* | *IV* | *flush/dysp/shock* | *---* | *1 mo* | *11,8* | *nd* | *neg* |  | *Ibu, Met, Ox* | *DF* |
| 14 | M | 59 | po | ? | I | flush | hours | 1 mo | 3,71 | nd | neg | nd | nd | DF |
| *16* | *M* | *74* | *iv* | *?* | *III* | *prur/coll* | *dur inf* | *3 wk* | *131* | *nd* | *neg* |  | *AA, Ibu, Mef, Met, Ox* | *DF* |
| 17 | F | 42 | iv | yes | I | urt | 60 min | 1 mo | nd | nd | neg |  | ASA, AA, Mef, Met, Ox | DF |
| 18 | M | 63 | po | yes | I | urt | 60 min | 3 mo | nd | nd | neg |  | ASA, AA, Ibu, Mef, Met, Ox | DF |
| 19 | F | 47 | po | yes | I | prur | --- | 3 mo | nd | nd | neg |  | ASA, AA, Ibu, Mef, Met, Ox | DF |
| *20* | *F* | *47* | *po* | *yes* | *III* | *prur/naus/cramps/coll* | *30 min* | *2 mo* | *49,1* | *neg* | *neg* |  | *ASA, Ibu, Mef,* | *DF* |
| *22* | *F* | *67* | *po* | *?* | *III* | *urt/naus/cramps/diarr/coll* | *60 min* | *---* | *98,1* | *neg* | *neg* |  | *ASA, Mef, Met, Ox* | *DF* |
| 23 | M | 68 | im | yes | II | ang/dysp | 1-2 h | --- | nd | nd | neg |  | ASA, AA, Ibu, Mef, Met, Ox | DF |
| 24 | F | 22 | iv | ? | II | dysp/vert | dur inf | 2 d | 14,4 | neg | neg |  | ASA, Ind, Mef, Met, Ox | DF |
| 25 | F | 39 | po | yes | II | urt/dysp | 3 h | 2 mo | 175 | **pos** | neg |  | AA, Ibu, Mef, Met, Ox | DF |
| 28 | M | 61 | po | ? | I | prur | 1-2 h | 17 d | 30,7 | neg | neg | nd | nd | DF |
| 29 | F | 26 | iv | yes | II | urt/flush/dysp | 30 min | 7-8 wk | nd | nd | neg |  | ASA, AA, Ibu, Mef, Met, Ox | DF |
| 30 | M | 65 | po | yes | I | ang | 2-3 h | 1 d | 96,8 | neg | neg |  | ASA, AA, Ibu, Mef, Met, Ox | DF |
| *33* | *F* | *47* | *po* | *yes* | *III* | *dysp/coll* | *1-2 h* | *2 mo* | *275* | *neg* | *neg* |  | *ASA, Ibu, Mef, Met* | *DF* |
| *35* | *F* | *61* | *po* | *?* | *III* | *flush/dysp/hypo/coll* | *20 min* | *1-2 mo* | *948* | ***pos*** | *neg* |  | *AA, Ibu, Ind, Met, Ox* | *DF* |
| *36* | *F* | *36* | *po* | *?* | *III* | *urt/flush/cramps/dysp/tach/vom* | *2 h* | *3 d* | *238* | ***pos*** | *neg* | *nd* | *nd* | *DF, P* |
| *37* | *M* | *79* | *po* | *?* | *III* | *prur/coll* | *1 h* | *1 mo* | *11* | ***pos*** | *neg* |  | *ASA, AA, Ibu, Mef, Met* | *DF* |
| 38 | M | 67 | po | ? | II | ang/dysp | 30 min | 3-4 mo | 94,8 | neg | neg |  | ASA, Mef, Ox | DF, P |
| 39 | F | 68 | po | yes | II | prur/urt/ang/dysp | 5 h | 3 d | 85 | neg | neg |  | ASA, AA, Mef, Met, Ox | DF |
| 40 | F | 36 | iv | ? | II | naus/vert | 2 min | 18 d | 7,5 | neg | neg | nd | nd | DF |
| *42* | *F* | *48* | *po* | *?* | *IV* | *prur/vert/naus/vom/shock* | *5 min* | *4 d* | *126* | *neg* | *neg* |  | *ASA, AA, Ibu, Mef, Met, Ox* | *DF* |
| *44* | *F* | *66* | *po* | *yes* | *III* | *urt/coll* | *10-300 min* | *5 y* | *14,9* | *neg* | *neg* |  | *ASA, AA, Ibu, Mef* | *DF* |
| *45* | *M* | *50* | *po* | *yes* | *III* | *prur/naus/coll* | *30 min* | *1 mo* | *31,6* | *neg* | *neg* |  | *ASA, AA, Ibu, Mef, Met, Ox* | *DF* |
| 46 | F | 46 | po | yes | II | ang/dysp | hours | 3 wk | 72,8 | neg | neg |  | AA, Ibu, Mef, Met, Ox | DF |
| *47* | *M* | *71* | *po* | *?* | *III* | *prur/coll* | *90 min* | *1 mo* | *151* | *neg* | *neg* |  | *ASA, AA, Ibu, Ind, Mef, Met* | *DF* |
| *48* | *F* | *48* | *iv* | *yes* | *III* | *dysp/vert/coll* | *10 min* | *4 mo* | *11,7* | *neg* | *neg* |  | *ASA, AA, Ibu, Ind, Mef, Met* | *DF* |
| 50 | F | 42 | im | yes | II | prur/ang/dysp | 10 min | 3 mo | 2040 | **pos** | neg |  | ASA, AA, Ibu, Ind, Met, Ox | DF |
| *51* | *F* | *73* | *po* | *yes* | *III* | *urt/coll* | *3 h* | *5 mo* | *78,7* | *neg* | *neg* |  | *ASA, AA, Cel* | *DF* |
| *52* | *M* | *62* | *po* | *?* | *III* | *prur/urt/ang/naus/dysp/coll* | *<1 h* | *5-6 mo* | *101* | *neg* | *neg* | *DF* | *ASA, Ox* | *DF* |
| 53 | F | 69 | po | ? | I | prur/urt | ? | 2 y | 32,8 | neg | neg | DF | ASA, AA, Ox | DF, P |
| 58 | M | 38 | po | ? | II | prur/flush/ang/vert/hypo | 10 min | 3 mo | 22,1 | neg | neg | DF | ASA, AA | DF |
| 59 | F | 80 | po | yes | II | prur/ang/dysp | 60 min | 4 y | 18,9 | neg | **DF** | nd | nd | DF |
| NSAIDs patients – multiple NSAIDs-reactive | | | | | | | | | | | | | | |
| 4 | F | 66 | po | ? | I | ang | hours | 3 d | 106 | nd | neg | nd | nd | DF, Mef |
| 6 | M | 41 | po | ? | I | ang | hours | 1 mo | 517 | nd | neg | ASA |  | DF |
| 12 | M | 65 | po | ? | II | urt/ang/dysp | 5 min | 2 y | 96 | nd | neg | Met | AA, Ox | DF |
| 13 | F | 67 | iv | ? | II | flush/tach/vert | 30 min | 2 mo | 90,5 | nd | neg | nd | nd | DF, ASA |
| *15* | *M* | *50* | *cu* | *?* | *III* | *prur/ang/diarr* | *hours* | *---* | *350* | *nd* | *neg* |  | *AA, Mef* | *DF, ASA* |
| *21* | *F* | *54* | *po* | *yes* | *III* | *prur/ang/naus/coll* | *30 min* | *3 mo* | *80,1* | ***pos*** | *neg* |  | *AA, Ibu, Met, Ox* | *DF, Mef* |
| *26* | *F* | *50* | *po* | *?* | *III* | *prur/flush/ang/dysp/coll* | *3 h* | *12 d* | *302* | *neg* | *neg* |  | *AA, Ibu, Mef, Met, Ox* | *DF, ASA* |
| 27 | M | 52 | iv | ? | I | prur/urt | dur inf | 5 y | 56,7 | neg | neg | Mef, Ox |  | DF, ASA |
| 31 | M | 28 | po | yes | I | urt | 2 h | 26 d | nd | nd | **ASA, Ibu, Met, Ox** | Mef, Ox |  | DF, ASA, Ibu |
| 32 | F | 59 | po | yes | II | ang/dysp/vert | 2-3 h | 3-4 mo | nd | nd | neg | nd | nd | DF, ASA, Met |
| 34 | M | 51 | iv | yes | I | ang | 1-2 h | 3 wk | 184 | neg | neg | ASA |  | DF |
| 41 | F | 59 | po | yes | I | prur/urt | 1-2h | 3-4 wk | 118 | neg | neg | ASA, Ox |  | DF, AA, Ibu, Met |
| 43 | F | 66 | po | ? | I | prur/ang | 6 h | 6 mo | nd | nd | neg | Ibu |  | DF |
| 49 | F | 61 | po | yes | II | dysp/vert | 15 min | 3 mo | 11,3 | neg | neg | nd | nd | DF, AA, Ibu |
| 54 | F | 47 | po | ? | I | prur/flush | 30 min | years | 43,8 | **pos** | neg | DF | Ox, Nim | ASA, Mef |
| 55 | F | 34 | po | ? | II | urt/ang/dysp | ? | 3 y | 70,3 | **pos** | **DF** | DF, AA, Nim | ASA, Ox, Cel | Ibu |
| 56 | M | 34 | iv | ? | I | urt | ? | 1 y | 19,5 | neg | neg | DF | AA | DF, ASA, Mef |
| 57 | M | 63 | po | yes | I | urt/ang | ? | 3y | 54,6 | neg | neg | DF, ASA | Ox | DF |

Patients categorized as selectively DF-reactive versus multiple NSAIDs-reactive by history and oral provocation testing (OPT). Patients showing severe reactions (grade III/IV) in italic. Sens, potential sensitization due to prior intake; onset, clinical onset; inter, interval between incidence and clinical follow up; SX1, specific IgE against common aeroallergens; SPT, skin prick test; DHR, drug hypersensitivity reaction; route, route of application: iv, intraveneous, im, intramuscular, po, per oral; clinical symptoms: ang, angioedema, coll collapse, diarr, diarrhea, dysp, dyspnoe, hypo, hypotension, naus, nausea, prur, pruritus, tach, tachycardia, urt, urticaria, vert, vertigo; NSAIDs: AA, acetaminophen, ASA, acetylsalicylic acid, Cel, celebrex, Ibu, ibuprofen, Mef, mefenamic acid, Met, metamizole, Nim, nimesulid, Ox, oxicams; results above threshold in bold.
