# Supplementary material for: Diclofenac Hypersensitivity: Antibody Responses to the Parent Drug and Relevant Metabolites
Source: PLoS One. 2010 Oct 28;5(10):e13707. doi: 10.1371/journal.pone.0013707 (PMC2965666; doi:10.1371/journal.pone.0013707)
Supplement: Table S2 — Combined results of BAT and CAST analyses (0.13 MB DOC) [file pone.0013707.s002.doc]

**Table S3.** Combined results of BAT and CAST analyses

|  |  |  | BAT | Stimulation controls | | | Hapten-HSA conjugate | | | | | | | |
| --- | --- | --- | --- | --- | --- | --- | --- | --- | --- | --- | --- | --- | --- | --- |
| ID | Group | Grade | CAST | BSB | fMLF | α-FcεRI | DF | DF5der | 3'OH-DF | 4'OH-DF | 5OH-DF | 4'5diOH-DF | 3'OH4metO-DF | mock |
| *#20* | *DF* | *III* | *% CD63^high^* | *1.2* | ***28.9*** | *2.0* | *1.9* | *n.d.* | *0.8* | *1.2* | *1.4* | *2.9* | *1.3* | *0.7* |
|  |  |  | *sLT pg/ml* | *72* | *n.d.* | ***212*** | *61* | *n.d.* | *59* | *52* | *<50* | *<50* | *<50* | *73* |
| #22 | DF | III | % CD63^high^ | 2.7 | **32.8** | **19.3** | 5.0 | n.d. | 1.4 | 3.5 | 4.6 | 3.3 | 3.4 | 2.1 |
|  |  |  | sLT pg/ml | <50 | n.d. | **657** | <50 | n.d. | <50 | <50 | <50 | <50 | <50 | <50 |
| #30 | DF | I | % CD63^high^ | **8.1** | **39.2** | **21.2** | **6.2** | n.d. | **6.8** | **5.2** | n.d. | **7.3** | **5.8** | **6.1** |
|  |  |  | sLT pg/ml | 90 | n.d. | **330** | 70 | n.d. | 70 | 75 | 85 | 120 | 110 | 120 |
| #33 | DF | III | % CD63^high^ | 2.7 | **49.1** | **84.5** | 2.2 | n.d. | 2.3 | 1.3 | 2.1 | 2.0 | 0.7 | 1.2 |
|  |  |  | sLT pg/ml | <50 | n.d. | **>3200** | <50 | n.d. | <50 | <50 | <50 | 65 | 65 | <50 |
| #37 | DF | III | % CD63^high^ | 3.0 | **55.6** | **59.3** | 0.1 | n.d. | 0.8 | 1.1 | 0.9 | 0.2 | 0.5 | 0.3 |
|  |  |  | sLT pg/ml | <50 | n.d. | **2064** | <50 | n.d. | <50 | <50 | <50 | <50 | 30 | <50 |
| #40 | DF | II | % CD63^high^ | 2.7 | **30.6** | **83.6** | 2.8 | n.d. | 2.6 | 2.2 | 2.6 | 2.8 | 3.6 | 3.5 |
|  |  |  | sLT pg/ml | 90 | n.d. | **>3200** | 120 | n.d. | 130 | 120 | 90 | 85 | 90 | 90 |
| #42 | DF | IV | % CD63^high^ | 4.5 | **67.4** | **24.3** | 5.4 | n.d. | 3.8 | 4.7 | 5.2 | 3.5 | 2.9 | 4.5 |
|  |  |  | sLT pg/ml | <50 | n.d. | **480** | 95 | n.d. | 95 | 80 | <50 | <50 | <50 | 80 |
| #45 | DF | III | % CD63^high^ | 1.7 | **20.3** | **55.8** | 1.4 | n.d. | 0.5 | 0.6 | 1.0 | 1.0 | 1.4 | 1.1 |
|  |  |  | sLT pg/ml | 85 | n.d. | **660** | 90 | n.d. | <50 | 85 | 70 | <50 | <50 | <50 |
| #46 | DF | II | % CD63^high^ | 4.8 | **63.0** | **61.4** | 3.3 | n.d. | 2.9 | 2.9 | 3.0 | 3.3 | 2.6 | 2.0 |
|  |  |  | sLT pg/ml | <50 | n.d. | **1600** | <50 | n.d. | <50 | <50 | <50 | <50 | <50 | <50 |
| #47 | DF | III | % CD63^high^ | 3.7 | **55.0** | **19.3** | 2.6 | n.d. | n.d. | n.d. | n.d. | n.d. | n.d. | 1.7 |
|  |  |  | sLT pg/ml | n.d. | n.d. | **n.d.** | n.d. | n.d. | n.d. | n.d. | n.d. | n.d. | n.d. | n.d. |
| #50 | DF | II | % CD63^high^ | 1.5 | **65.7** | **36.2** | 1.6 | n.d. | 0.5 | 0.8 | 0.9 | 1.5 | 1.0 | 0.9 |
|  |  |  | sLT pg/ml | 55 | n.d. | **688** | 71 | n.d. | <50 | 59 | 95 | 67 | <50 | <50 |
| #52 | DF | III | % CD63^high^ | 2.1 | **46.2** | **27.7** | 2.3 | 1.9 | 0.9 | 1.3 | 1.9 | 0.7 | 1.9 | 2.2 |
|  |  |  | sLT pg/ml | 84 | n.d. | **849** | 156 | n.d. | 161 | 111 | 78 | 133 | 133 | 107 |
| #53 | DF | I | % CD63^high^ | 3.9 | **21.9** | **91.3** | 0.8 | 4.8 | 1.1 | 1.1 | 1.1 | 0.7 | 1.4 | 1.5 |
|  |  |  | sLT pg/ml | 68 | n.d. | **996** | 68 | n.d. | 55 | 59 | 81 | 55 | 63 | 73 |
| #58 | DF | II | % CD63^high^ | 1.8 | **69.3** | **70.8** | 1.1 | 3.3 | 1.5 | 1.3 | 1.8 | 1.6 | 1.9 | 0.9 |
|  |  |  | sLT pg/ml | 73 | n.d. | **>3200** | 124 | n.d. | **194** | 111 | 89 | 153 | 102 | 73 |
| #59 | DF | II | % CD63^high^ | 1.0 | **22.4** | **79.0** | 0.1 | n.d. | 0.9 | 0.6 | 0.9 | 1.0 | 1.0 | 1.2 |
|  |  |  | sLT pg/ml | n.d. | n.d. | **n.d.** | n.d. | n.d. | n.d. | n.d. | n.d. | n.d. | n.d. | n.d. |
| *#54* | *NSAIDs* | *I* | *% CD63^high^* | ***44.7*** | ***88.6*** | ***84.2*** | ***51.2*** | *n.d.* | ***54.9*** | ***52.7*** | ***60.4*** | ***52.7*** | ***54.7*** | ***51.7*** |
|  |  |  | *sLT pg/ml* | ***176*** | *n.d.* | ***>3200*** | *155* | *n.d.* | ***185*** | ***175*** | ***207*** | ***187*** | ***201*** | ***223*** |
| #55 | NSAIDs | II | % CD63^high^ | 1.8 | **60.6** | **97.4** | 0.4 | n.d. | 0.6 | 0.5 | 0.4 | 1.0 | 0.6 | 0.0 |
|  |  |  | sLT pg/ml | 90 | n.d. | **650** | 72 | n.d. | 79 | <50 | 66 | 94 | 95 | 133 |
| #56 | NSAIDs | I | % CD63^high^ | 1.4 | **64.0** | **82.0** | 1.2 | n.d. | 0.6 | n.d. | 3.8 | 1.8 | 1.5 | 1.9 |
|  |  |  | sLT pg/ml | 76 | n.d. | **2103** | 74 | n.d. | 76 | 72 | 76 | 94 | 98 | 94 |
| #57 | NSAIDs | I | % CD63^high^ | 1.9 | **42.0** | **49.9** | n.d. | n.d. | 2.8 | 2.3 | 1.1 | 1.6 | 0.7 | 1.5 |
|  |  |  | sLT pg/ml | 123 | n.d. | **1154** | 158 | n.d. | 137 | 120 | 136 | 154 | 126 | 105 |
| CG01 | control | 0 | % CD63^high^ | 4.7 | **82.2** | **68.5** | 3.7 | n.d. | 4.1 | 3.7 | 4.6 | 4.7 | 4.8 | 4.9 |
|  |  |  | sLT pg/ml | 90 | n.d. | **1528** | <50 | n.d. | <50 | <50 | <50 | <50 | <50 | 78 |
| CG02 | control | 0 | % CD63^high^ | 0.4 | **71.2** | **20.9** | 1.1 | n.d. | 0.6 | 1.2 | 0.9 | 0.9 | 0.4 | 0.4 |
|  |  |  | sLT pg/ml | 77 | n.d. | **659** | 53 | n.d. | 109 | 123 | 99 | 101 | 72 | 65 |
| CG03 | control | 0 | % CD63^high^ | 1.8 | **26.8** | **63.3** | 1.5 | n.d. | 1.6 | 1.4 | 1.6 | 2.1 | 1.4 | 1.3 |
|  |  |  | sLT pg/ml | 131 | n.d. | **>3200** | 158 | n.d. | 103 | 127 | 70 | 122 | 85 | 141 |

BAT, basophil activation test; CAST, cellular antigen stimulation test; BSB, basophil stimulation buffer as negative control; fMLF, formyl-Met-Leu-Phe as positive control; in italic: patients #20 and #54 were excluded due to nonresponsiveness to α-FcεRI or high background values, respectively; results above threshold in bold; patient #30 negative although above threshold (>5%) as stimulation index <2.
